# Essential (Mg, Fe, Zn and Cu) and Non-Essential (Cd and Pb) Elements in Predatory Insects (*Vespa crabro* and *Vespa velutina*): A Molecular Perspective

**DOI:** 10.3390/ijms22010228

**Published:** 2020-12-28

**Authors:** Giulia Andreani, Enea Ferlizza, Riccardo Cabbri, Micaela Fabbri, Elisa Bellei, Gloria Isani

**Affiliations:** 1 Department of Veterinary Medical Sciences, Alma Mater Studiorum—University of Bologna, via Tolara di sopra 50, Ozzano dell’Emilia, 40064 Bologna, Italy; giulia.andreani2@unibo.it (G.A.); rccabbri@gmail.com (R.C.); micaela.fabbri@unibo.it (M.F.); gloria.isani@unibo.it (G.I.); 2 Department of Experimental Diagnostic and Specialty Medicine, Alma Mater Studiorum-University of Bologna, via Belmeloro 8, 40126 Bologna, Italy; 3 Department of Surgery, Medicine, Dentistry and Morphological Sciences with Transplant Surgery, Oncology and Regenerative Medicine Relevance, Proteomic Lab, University of Modena and Reggio Emilia, via del pozzo 71, 41124 Modena, Italy; elisa.bellei@unimore.it

**Keywords:** Asian yellow-legged hornet, European hornet, electrophoresis, proteomics, iron, copper, zinc, magnesium, cadmium, lead

## Abstract

The recent introduction of the Asian yellow-legged hornet, *Vespa velutina,* into Europe has raised concern regarding the threat to honeybees and the competition with the European hornet, *Vespa crabro*. The aim of this study was to investigated essential (Mg, Fe, Zn, Cu) and non-essential (Cd and Pb) elements in these two species. Element concentrations were determined in the whole body and separately in the head, thorax and abdomen using atomic absorption spectrometry (AAS). The changes in essential element concentration and speciation during metamorphosis were also studied using size exclusion chromatography followed by AAS and proteomic analysis. In both species, the essential elements were more concentrated in the abdomen due to the presence of fat bodies. Magnesium, Fe and Zn concentrations were significantly higher in *V*. *crabro* than in *V. velutina* and could have been related to the higher aerobic energy demand of the former species required to sustain foraging flight. Low concentrations of Cd and Pb were indicative of low environmental exposure. The concentration and speciation of essential elements, particularly Fe, varied among the developmental stages, indicating a modification of ligand preferences during metamorphosis. Overall, the results in the present study provide a better understanding of the hornet metal metabolism and a foundation for additional studies.

## 1. Introduction

Insects, which are characterized by a wide variety of physiological and biochemical peculiarities, can offer interesting models for the study of essential element metabolism, despite their phylogenetic distance from vertebrates. As an example, a recent study carried out on *Drosophila melanogaster* has highlighted a complex network of proteins regulating Fe homeostasis and has pointed out that Fe biochemistry and regulation is conserved between vertebrates and *Drosophila*, with the exception of the transferrin receptor [1].

Hornets, the largest of the eusocial wasps, belong to the genus *Vespa* and are at the top of the food chain, feeding primarily on other insects. They live in large colonies, producing thousands of sterile workers over the lifetime of the colony and hundreds of fertile drones and queens towards the end of the season. In species living in temperate zones, such as the European hornet *Vespa crabro* and the Asian yellow-legged hornet *Vespa velutina*, the fertilized queens found new colonies alone the following spring [2]. This mechanism of founding colonies has facilitated the spread of these species through human trade. In fact, *V. crabro* has been introduced into North America [3] while *V. velutina* has been introduced into Europe [4]. As a consequence, and in accordance with European Union Regulations EU 1143/2014 [5] and EU 1141/2016 [6], the Asian yellow-legged hornet has been included in the “blacklist” of invasive alien species of Union concern. Regarding these species, EU member states must “prevent their introduction, implement early warning and rapid response systems, eradicate or control their spread or manage those species which are already widespread in their territory” [5,6]. Understanding the biology of this invasive species in comparison with the native European hornet is therefore of pivotal importance to contrast its spread over the territory and limit the competition to native hornet species and the threats to honeybees, which are the preferred prey of this predator insect [4].

Essential and non-essential element concentrations in honeybees and wasps have been widely used as indicators of environmental pollution [7]. However, essential elements, in particular Mg, Fe, Zn and Cu, carry out different biochemical functions related to cell metabolism and regulation, and their concentrations are controlled by sophisticated homeostatic mechanisms which include uptake, transport, intracellular distribution and excretion [8]. Therefore, their concentrations are intimately connected with the main physiological cell functions and can be used as a tool for obtaining information regarding the biochemical adaptations of an organism. The determination of element concentrations and the characterization of element-binding biomolecules require specific analytical techniques. The study of metal-binding proteins can be addressed by hyphenated techniques combining a chromatographic fractionation and electrophoretic separation of proteins with sensitive element detection in the fractions obtained [9]. A canonical and widely used approach for the study of these proteins includes size exclusion chromatography and 1(2)-D gel electrophoresis for protein fractionation as a function of molecular size coupled with inductively coupled plasma mass spectrometry (ICP-MS) or atomic absorption spectrometry (AAS) for metal quantification.

This study originally arose from the need to deepen the knowledge regarding *V. velutina* in relation to its competition with *V. crabro*. The first aim of this study was to determine the concentrations of four essential (Mg, Fe, Zn, Cu) and two non-essential (Cd, Pb) elements in adult specimens to highlight any differences between the two species. For this purpose, the concentrations of the elements selected were analyzed not only in the whole body but also separately in its main anatomical components (head, thorax and abdomen). Subsequently, the collection of an entire nest of European hornets gave us the opportunity of obtaining larvae, pupae and adults from the same colony. Therefore, the second aim was to study in *V. crabro* the element profile and speciation during the organism development using size exclusion chromatography coupled with AAS. Finally, some of the most abundant cytosolic proteins were separated and identified using a canonical proteomics approach based on SDS–PAGE electrophoresis and mass spectrometry to shed more light on the biochemical adaptations and possibly identify any correlation with element concentrations.

## 2. Results

### 2.1. Essential and Non-Essential Elements

The concentrations of Mg, Fe, Zn, Cu, Cd and Pb in *V. crabro* and *V. velutina* are reported, according to their abundance in the samples analyzed, in Table 1. In general, of the essential elements, Mg was the most abundant while Cu was the least abundant. Cadmium and Pb were present at detectable concentrations in all the samples analyzed. In adults, the abdomen accumulated the highest concentrations of the elements, with the exception of Pb in *V. crabro* and Mg in *V. velutina*. However, the highest metal concentrations, in particular for Cd, which reached a mean concentration as high as 296.2 ng/g, were determined in the meconium (Table 2), a fecal mass, which contains waste products, including environmental contaminants, and is excreted by the larvae on the floor of the cell when they become pupae.

Magnesium presented significantly higher concentrations in *V. crabro* (378.16 ± 70.12 µg/g) than in *V. velutina* (300.62 ± 58.69 µg/g) (*p* < 0.01). When considering the different body parts, Mg concentrations were significantly higher in the head and abdomen than in the thorax in *V. crabro*, and in the head and thorax than in the abdomen in *V. velutina,* while *V. crabro* showed significantly higher concentrations of Mg in the head and abdomen (*p* < 0.01) than *V. velutina*. Regarding the other samples, significantly lower Mg concentrations were found in the queens, larvae and pupae than in the adults of *V. crabro* (*p* < 0.05). The queens also showed lower concentrations of Mg than the larvae and pupae (*p* < 0.05).

In the whole bodies of the adults, significantly higher concentrations of Fe were present in *V. crabro* (70.61 ± 18.02 µg/g) than in *V. velutina* (46.66 ± 7.27 µg/g) (*p* < 0.01). Iron was accumulated preferentially in the abdomen, which had values significantly higher than in the head and thorax in both *V. crabro* (*p* < 0.01) and *V. velutina*; in turn, the Fe levels were significantly higher in the thorax than in the head (*p* < 0.01) only in *V. velutina*. Between the two species, Fe was significantly more concentrated in the head of *V. crabro* (*p* < 0.01) and in the thorax of *V. velutina* (*p* < 0.05). Significantly lower concentrations (*p* < 0.05) were also determined in the queens, pupae and larvae than in the adult specimens of *V. crabro*.

The concentrations of Zn detected in *V. crabro* (35.62 ± 6.97 µg/g) were significantly higher than in *V. velutina* (23.98 ± 3.93 µg/g) (*p* < 0.01); however, in both species, the Zn distribution in the body parts was characterized by the following order of concentrations abdomen > head > thorax with all the comparisons showing statistically significant differences (*p* < 0.01). The highest Zn concentrations were detected in the queens and adults, with significant differences (*p* < 0.05) with respect to the pupae and larvae. Moreover, the adults also presented higher Zn concentration than the larvae and pupae (*p* < 0.01).

The Cu concentrations were not statistically different between *V. crabro* (11.79 ± 2.10 µg/g) and *V. velutina* (12.67 ± 2.48 µg/g), with a similar distribution in the body parts characterized by the following order: abdomen > thorax > head with significant differences among the body parts (*p* < 0.01). When comparing Cu levels between the two species in the different body parts, only the abdomen of *V. velutina* was significantly richer in Cu than the abdomen of *V. crabro* (*p* < 0.01). Queens had Cu concentrations not significantly different from those detected in adults, while both adults and queens showed significantly higher Cu concentrations (*p* < 0.05) than the larvae and pupae.

Regarding the non-essential elements, Cd concentrations were higher, albeit not significantly different, in *V. velutina* (61.70 ± 25.27 ng/g) than in *V. crabro* (44.92 ± 22.51 ng/g). The Pb concentrations were significantly (*p* < 0.01) higher in *V. crabro* (101.00 ± 94.67 ng/g) than in *V. velutina* (30.91 ± 17.76 ng/g). In both species, the Cd and Pb concentrations in the body parts showed the following decreasing order: abdomen > head > thorax, except for Pb, which in *V. crabro* reached the highest concentrations in the head (128.20 ± 2.54 ng/g).

### 2.2. Size Exclusion Chromatography and Element Speciation

The element distribution between the pellet and the cytosolic fractions obtained after centrifugation of the samples from the adults, pupae and larvae of *V. crabro* was determined and is reported in Table 3. In the adults, Mg was significantly (*p* < 0.05) more concentrated in the pellets than in the cytosolic extracts while, in the larvae and pupae, it was significantly (*p* < 0.05) more concentrated in the cytosolic extracts. The Fe in the larvae was mainly present in the cytosolic fraction; however, in the pupae and adults, a significantly higher percentage was present in the pellets (*p* < 0.05). Zinc was uniformly distributed between the two fractions in the adults and pupae while, in the larvae, the cytosolic extract retained significantly more of this metal than the pellets. Copper in the adults was also not significantly different between the pellets and cytosols while, in the larvae and pupae, it was present in a significant (*p* < 0.05) greater percentage in the cytosols.

**Figure 1:**
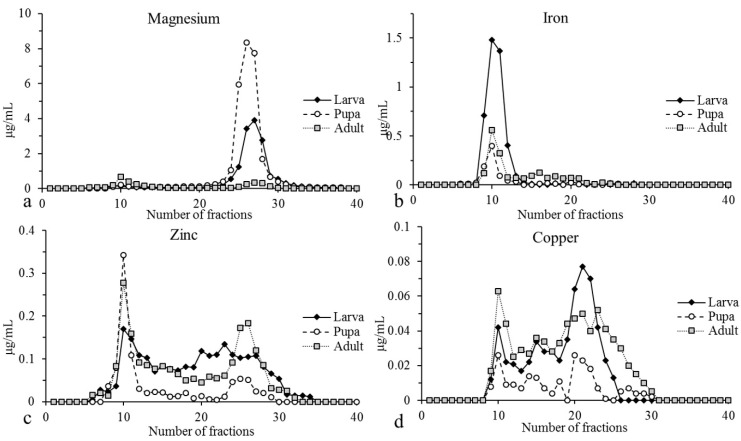
Element profiles of the cytosolic extracts obtained from the larvae, pupae and adults after size exclusion chromatography. The element concentrations are expressed in µg/mL.

Element profiles from the cytosolic extracts of the larvae, pupae and adults after gel filtration chromatography are reported in Figure 1. Magnesium and Fe presented completely different elution profiles; in the cytosolic extracts, the greater part of the Mg appeared in fractions corresponding to the free metal ions (fractions 24–30), while Fe was bound to ligands with high molecular mass (HMM) (fractions 9–12). However, Zn and Cu were spread throughout the chromatograms; both metals presented a first peak corresponding to HMM ligands, followed by other less consistent peaks in all the developmental stages, with the exception of Cu in the larvae cytosolic extracts in which the bulk of the metal was bound to low molecular mass (LMM) ligands (fractions 20–22).

### 2.3. SDS–PAGE and Protein Identification Using Mass Spectrometry

**Figure 2:**
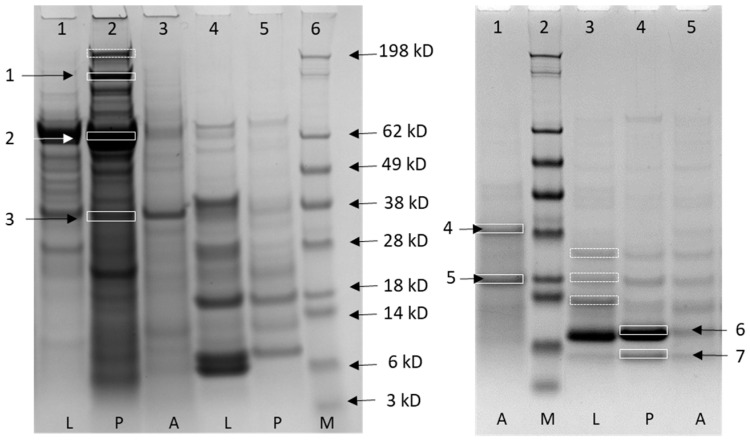
Representative gels (4–12%, Coomassie staining) of fractions obtained after size exclusion chromatography of cytosolic extracts of larvae, pupae and adults of European hornet. Left: lanes 1–3, fraction 10 from larvae (L), pupae (P) and adults (A), respectively; lanes 4–5, fraction 15 from larvae and pupae, respectively; lane 6, molecular mass marker (M) (kD). Right: lane 1, fraction 15 from adults; lane 2, molecular mass marker; lanes 3–5, fraction 19 from larvae, pupae and adults, respectively. Continuous line rectangles indicate bands excised and analyzed by mass spectrometry that gave protein identifications (Table 4). Dotted line rectangles indicate bands excised and analyzed by mass spectrometry that did not result in protein identification.

The fractions containing the most relevant metal burden, namely fractions 10, 11, 15, 18, 20 and 21, underwent SDS–PAGE. Fractions over twenty-two were excluded because they no longer contained proteins, but only small peptides, free amino acids and free ions. A representative gel is reported in Figure 2. Thirteen proteins were successfully identified from the 11 bands excised from the gels. No proteins have been identified in four bands. The proteins identified and their functions are reported in Table 4 and Table S1. Eight were enzymes involved in the carbohydrate metabolism, namely 4-alpha-glucanotransferase, alpha-glucosidase isozyme, trehalase, glucose-6-phosphate isomerase, phosphoglucomutase 2, triosephosphate isomerase, glyceraldehyde-3-phosphate dehydrogenase and enolase. The other proteins were sodium channel protein, endoplasmic reticulum chaperone BiP, dynein light chain 2, Cu-Zn superoxide dismutase and ubiquitin-like domain-containing protein.

The biological processes and molecular functions related to these proteins are reported according to gene ontology (GO) and UniProt in Supplementary Table S1. The proteins identified were involved in different molecular functions, mainly enzymatic (e.g., hydrolase or isomerase activity) and binding (e.g., metals, adenosine triphosphate [ATP] or guanosine triphosphate [GTP], carbohydrates) activities. The most common biological processes involved carbohydrate metabolism, glycolytic activity or gluconeogenesis; two proteins were involved in a microtubule-based process and two proteins in the transport of ions or proteins. For three proteins, the biological process is still unknown.

## 3. Discussion

Data regarding essential and non-essential element concentration and metabolism in hornets are fragmentary; therefore, the present results will primarily be discussed in the framework of knowledge obtained in other Hymenoptera, namely honeybees and wasps and, in some cases, with data reported for *Drosophila*, which is the most studied species of insects. In living organisms, the tissue concentrations of Mg, Fe, Zn and Cu are the results of sophisticated biochemical mechanisms shaped by evolution to cope with specific physiologic and metabolic peculiarities and with a changing environment. Consequently, the differences in element concentrations and the metabolic adaptations to maintain homeostasis can be due to a plethora of mechanisms; the most relevant will be discussed in the following paragraphs. On the other hand, tissue concentrations of the non-essential elements Cd and Pb are the result of a life-long accumulation and will be discussed as the result of possible environmental contamination.

### 3.1. Essential and Non-Essential Elements

In hornets, the concentrations of the essential elements analyzed fell into the range of those reported in specimens of the common wasp *Vespula vulgaris* [10], with the exception of Zn, the concentrations of which were lower in hornets with respect to wasps. Regarding the comparison with honeybees, higher concentrations of Fe, Zn and Cu were determined in the hornets with respect to the honeybees collected at 35 sites in Central Italy [11]. Although a similar pattern of exploratory behavior and a partial overlapping of feeding preferences have been reported in workers of *V. crabro* and *V. velutina* [12], the data reported in the present study showed higher concentrations of Mg, Fe and Zn in the whole body of *V. crabro* with respect to *V. velutina*. Respirometry studies conducted on *A. mellifera* and other bee species have supported the notion that carbohydrates are used as the primary fuel for muscles [13], and Mg and Fe are the most important cofactors of the glycolytic enzymes and complexes of the respiratory chain, respectively. It can be hypothesized that the higher concentrations of Mg and Fe in *V. crabro* might be related to a higher energy demand required to sustain the flight of a heavier species rather than flight at a higher speed. In fact, the mean flight speed for *V. velutina* workers determined in a flight mill was 1.56 ms^−1^ [14], similar to 1.86 ms^−1^ reported by Spiewok and Schmolz for the European hornet [15]. Moreover, recent research tracked the flight of *V. velutina* in the field using an innovative harmonic radar; the measured speed was approximately 5 ms^−1^, with a maximum of 6.9 ms^−1^ [16], which is similar to the data reported for free-flying European hornets (about 6 ms^−1^) [15]. Therefore, these data do not support the hypothesis of different flight speeds and other physiological and environmental factors may be involved in determining the higher concentrations of Mg and Fe in *V. crabro*.

The concentrations of Pb and Cd determined in adult hornets in the present study were lower than those reported by other authors regarding the paper wasp *Polistes dominulus* [17,18] and the common wasp *V. vulgaris* [10] living in environments with different anthropic impacts, confirming that the hornets sampled lived in areas characterized by low environmental Pb and Cd pollution. However, the significantly higher Pb concentrations measured in *V. crabro* than in *V. velutina* may have been linked to the different levels of environmental Pb pollution in the sampling areas. The specimens of *V. velutina* were sampled in a rural area while the specimens of *V. crabro* were collected in an area scarcely anthropized but crossed by a very busy road. Accordingly, in *P. dominulus* from a southeastern area of Spain, wasps from urban sites had double the Pb concentration of wasps from rural sites [17]. Comparable results were obtained in workers of the same species sampled in urbanized and rural areas in Italy, reporting that adult wasps living in an urbanized area (Florence) had a mean Pb concentration 3.4 times higher than that of the rural wasps [18]. The highest concentrations of Cd and Pb were determined in meconium. Larval fecal masses can be considered an interesting biological sample for environmental monitoring, as suggested by Urbini et al. (2006) [18], who used the meconium of *P. dominulus* to differentiate zones with different degrees of Pb pollution.

The impact of *V. velutina* on Hymenoptera other than honeybees is still relatively unexplored. On the basis of laboratory studies, Cini et al. (2018) [12] suggested that *V. velutina* might represent a potential competitor for the native European hornet due to a similar pattern of exploratory profile and overlapping of prey preference. On the other hand, a recent study on interspecific hierarchies reported that *V. crabro* is able to outperform *V. velutina* in laboratory conditions [19]. Finally, the effect of the invasion of *V. velutina* on the abundance of *V. crabro* population in a natural environment was analyzed for the first time by Carisio et al. 2020 [20]. The study, carried out in western Liguria (Italy), revealed that the detrimental effects of *V. velutina* on *V. crabro* are negligible, and a consistent population of the latter can be considered as an ecological barrier slowing down the diffusion of yellow-legged hornet in Italy. The data reported in the present paper have revealed similar patterns of essential elements, suggesting common mechanisms of handling and homeostasis in the two species. However, quantitative differences were found. The higher concentrations of Mg, Fe and Zn determined in *V. crabro* associated with higher Pb and Cd concentrations may be due to a different environmental exposure. In fact, *A. mellifera* and other Hymenoptera are considered interesting biomonitor organisms that have been used to assess element distribution in different environments worldwide [7,10,18].

### 3.2. Factors Affecting Element Concentration and Metabolism

Analysis of the separate body parts revealed the relative distribution of Mg, Fe, Zn and Cu, mirroring the biochemical peculiarities of the different organs. In both species, the abdomen accumulated the highest concentrations of the elements analyzed, with the exception of Mg in *V. velutina*, due to the anatomical organization and the physiological functions of the organs present inside. While the thorax contains primarily wing muscles and the head hosts the brain, the abdomen is characterized by the presence of the viscera, including the fat bodies, the metabolic function of which is similar to that of the vertebrate liver [21]. The differential elemental distribution is particularly evident for Fe. The presence of Fe in the fat body has previously been reported in honeybees [22]. Using a multimodal imaging and analysis approach, Shaw et al. (2018) [22] demonstrated that abdominal Fe is accumulated in the trophocytes of the fat body, reaching high concentrations five days after eclosion. Whether the Fe present in the fat body is involved in magnetoreception or is a store of excess dietary intake is still an open question. The high concentrations of Fe found in the present study in the abdomens of both *V. crabro* and *V. velutina* are in accordance with the data reported in another species of hornet (*Vespa affinis*), which is able to accumulate Fe in the trophocytes localized in the abdomen [23].

Essential element metabolism has been scarcely investigated in relation to insect metamorphosis, a fascinating and complex biological process, which goes from eggs to adults through different stages of development. The authors found that metamorphosis was another prominent factor affecting element concentration and speciation, probably due to different food sources and the metabolic demand characterizing larvae, pupae and adults. The element concentrations determined in this study were significantly higher in adults with respect to those measured in larvae and pupae. Accordingly, in the mealworm beetle *Tenebrio molitor* the concentrations of Fe, Cu, and Zn were significantly higher in first-generation adults than in other stages [24]. In addition, it has been reported that the Fe concentration in worker honeybees increased with age from 0.07 µg/mg at eclosion to 0.19 µg/mg in 25 day-old honeybees [22], also suggesting an increasing trend in adult honeybees.

Studies on element speciation, i.e., the distribution of the element among defined chemical species in a biological system, are rare in insects, and the approach we used to investigate element speciation during insect ontogenesis has never been performed before. The changes in larvae, pupae and adults were addressed by non-denaturing size exclusion chromatography of the cytosolic extracts followed by element analysis of the chromatographic fractions using AAS. Because different molecular species are present in each chromatographic fraction, an additional separation step using SDS–PAGE electrophoresis was applied before protein identification with mass spectrometry. The coupling of size exclusion chromatography and multi-elemental detection using sensitive techniques is a consolidated approach for a semiquantitative screening for the presence of metal–biomolecule complexes in biological samples and has been widely used [9,25,26,27]. Of the developmental stages, an interesting difference in subcellular distribution and element speciation was recorded for Fe. In larvae, Fe was more concentrated in the cytosolic extracts than in the pellets. Iron, as the ferrous ion (Fe^2+^), is highly reactive, while the ferric ion (Fe^3+^) is less reactive; consequently, Fe-chaperones and transporters prevent the accumulation of free ferrous ions, which can react with lipids, proteins and other biomolecules, causing harmful oxidative damage [28]. Accordingly, in the elution profiles, the bulk of the Fe was bound to proteins, in particular to HMM proteins corresponding to fractions between 9 and 12. Surprisingly, ferritin was not identified in these fractions. Ferritin is the ubiquitous Fe storage molecule in many organisms, including insects. However, in contrast to vertebrates, in *D. melanogaster*, ferritins are not confined to the cell cytosol but are secreted into the hemolymph; they are responsible for dietary Fe intake from the midgut cells and for its delivery to the other tissues [29]. In this complex scenario, the paucity of cytosolic intracellular stores of ferritin may have led to the lack of its identification using mass spectrometry, and other iron-binding proteins present in the HMM peak remained uncharacterized. During metamorphosis, a redistribution of Fe between the soluble and insoluble fractions took place depending on the metabolic needs of the different developmental stages. In fact, in the pupae and adults, the Fe was more concentrated in the pellets than in the cytosolic extracts. In the pellets, Fe can be present as bioinorganic Fe in granules and, therefore, in an insoluble form [22] or bound to proteins contained inside nuclei or subcellular organelles, mainly mitochondria [30]. Accordingly, in honeybees, it has been reported that bioinorganic Fe granules are present in trophocytes only after the adults emerge from the comb [31].

In chromatograms of hornet cytosolic extracts, Zn and Cu were spread throughout the fractions, indicating an affinity for different molecular ligands. The identity of some of these ligands can be hypothesized from the position of the peak in the chromatogram or by means of direct identification using mass spectrometry [9]. Under the experimental conditions adopted in this study, the presence of Zn and Cu peaks at fractions 14–15, corresponding to an apparent MM of 30–32 kDa, could be considered indicative of the presence of Cu-Zn superoxide dismutase (SOD) [32], a cytosolic enzyme made up of two identical subunits of 16 kDa. In fact, this protein was identified using mass spectrometry in band 9, which was obtained after SDS-PAGE fractionation of the proteins present in fraction 15. The role of this enzyme as an antioxidant defense during prolonged flight and as a crucial anti-aging molecule has been studied in *Drosophila* and honeybees [33]. In flies, the overexpression of Cu-Zn SOD and catalase determined a one-third extension of the lifespan [34] while, in forager honeybees, Margotta et al. (2018) [35] suggested that oxidative damage due to decreased SOD activity in flight muscle contributed directly to organism senescence.

The presence of an LMM peak containing Cu and Zn and sometimes also Cd is a common finding in chromatograms from vertebrate and invertebrate cytosolic extracts [36,37]. This peak is due to metallothioneins (Mts), ubiquitous, cysteine-rich proteins of 6000–7000 D, playing an essential role in metal homeostasis and detoxification in eukaryotes, from yeast to mammals [38]. The Cu-containing peak at fractions 20–22 might therefore have been related to Mt. A gene encoding a putative Mt (AmMT) has recently been identified in *A. mellifera* [39], suggesting a role in metal binding and detoxification, also in Hymenoptera, for these proteins. The genome of *D. melanogaster* contains at least five Mt loci (*MtnA, MtnB, MtnC, MtnD* and *MtnE*) [40], and these five Mts show low affinity for Zn and high affinity for Cu and Cd [41,42]. The absence of a Zn-containing peak at fractions 20–22 in the Zn elution profile of hornet extracts is therefore intriguing and offers additional evidence for the metal-binding preference of insect Mt. The data obtained in the present study are also in accordance with the conclusion of Navarro and Schneuwly (2018) [43] that fly Mts act primarily as Cu-thioneins in comparison to their mammalian counterparts which are predominantly Zn-thioneins. The tight relationship between Mt expression and Cu accumulation is particularly evident in *Drosophila* larvae, which contain the so-called copper cells in the midgut [41]. The presence of these cells, also in hornet larvae, might be the cause of the higher Cu peak in larvae with respect to adults and pupae.

### 3.3. Proteins Identified

Eight of the 13 proteins unambiguously identified using mass spectrometry were enzymes involved in carbohydrate metabolism, in particular in glucose mobilization and catabolism. In insects, glucose is stored in two different metabolic forms: disaccharide trehalose, synthesized in the fat body and released into the hemolymph, and glycogen, synthesized and stored mostly in muscles and in the fat body [44]. Three enzymes involved in glucose mobilization were identified in the cytosolic extracts of the hornets. The glycogen debranching enzyme has two distinct active sites for its 4-alpha-glucanotransferase and amylo-alpha-1,6-glucosidase activities. This enzyme, which removes α-(1–6) linkages, detaches from glycogen units of glucose and is important for continued glycogen breakdown during insect flight [45]. Alpha-glucosidase catalyzes the hydrolysis of α-glucosidic bonds, releasing free glucose; this enzyme is present as a soluble form in the midgut lumen or bound to cell membranes and is important for the digestion of sucrose [46,47]. Trehalase is the enzyme that hydrolyses trehalose, a circulating disaccharide containing two D-glucose units in an α,α-(1-1) linkage, which plays a crucial role as a metabolic source of energy [48]. The other five enzymes, glucose-6-phosphate isomerase, triosephosphate isomerase, glyceraldehyde-3-phosphate dehydrogenase, phosphoglycerate mutase 2 and enolase, are all involved in glycolysis, the most relevant cytosolic catabolic pathway. The abundance of these enzymes can be related to either diet or to the need to fuel flight. As regards the dietary specialization, the adults of *V. crabro* feed predominantly on carbohydrates while the larvae feed on animal proteins [49]. Insect flight is the most energy-demanding exercise known, and the metabolic rate can increase 50-to 100-fold during exploratory flights [50]. The predominance of the enzymes of the carbohydrate catabolism is in accordance with the fact that worker honeybees rely on carbohydrates to sustain flight during foraging [13] and require high concentrations of Mg and Fe, which are the metal coenzymes of the enzymes involved in glycolysis, the Krebs cycle and the respiratory chain, as previously discussed. Moreover, the high intensity of the aerobic metabolism exposes cells to the risk of oxidative stress, mainly due to the production of superoxide radicals; the identification of Cu-Zn SOD in the cytosolic extracts, particularly abundant in those obtained from adults, is suggestive of the need for antioxidant defenses to cope with the oxidative stress during flight.

## 4. Materials and Methods

### 4.1. Sample Collection

European hornets were collected in an area located in Loiano, in the Tuscan-Emilian Apennines (Bologna District), Northern Italy, characterized by typical deciduous montane forests and by a temperate-cool climate. The samples of adult specimens, workers and queens, were obtained using bottle traps in green areas, with beer as bait, in the dry period (from July to September) while the larvae, pupae and meconium were collected directly from nests built in wooden sheds for agricultural tools. Caste discrimination was achieved according to the size and weight of the adult insects; specimens weighing more than 0.6 g and with a total body length higher than 2.5 cm were identified as queens, otherwise as workers. Two holometabolous stages were identified based on morphology. Larvae lack the body appendages of adults and are stored in open cells of the comb, while pupae are in a non-feeding, quiescent state inside a thick cocoon that protrudes from the comb. Insects fully pigmented inside the cocoon, and thus close to eclosion, were discarded.

The Asian yellow-legged hornets were sampled in agricultural areas, in the vicinity of hives kept in apiaries located in Bordighera (Imperia District). The collection of Asian yellow-legged hornets was part of a project aimed at containing the spread of *V. velutina*. The capture of workers present in front of the hives was carried out using clean entomological nets.

After the collection, each insect was placed in a single polyethylene vial, which was held in a cold storage container filled with dry ice (−78 °C). The animals were euthanized by freezing at −78 °C. Subsequently, they were brought to the laboratory where the specimens were carefully washed by rinsing them, sequentially, in two separate containers filled with deionized water, identified, checked for parasites and finally stored at −80 °C until analysis.

No permits from ethical committees were needed to sample the European and Asian yellow-legged hornets since they do not benefit from legal protection in Italy.

### 4.2. Metal Analysis

To avoid contamination, all the reagents were handled carefully; polyethylene disposables were thoroughly washed with HCl 1 N under a fume hood, and disposable gloves were worn during the procedure. All the reagents were from Merck (Darmstadt, Germany); the acids were of Suprapur grade. Samples (200–400 mg of wet tissue) were placed in individual acid-washed Teflon jars and were digested with 1–2 mL 65% HNO_3_ and 0.25–0.5 mL 30% H_2_O_2_ in a microwave oven for 5 min at 250 W, 5 min at 400 W, 5 min at 500 W and 1 min at 600 W. The cooled samples were transferred into 5–10 mL polyethylene volumetric flasks and were directly analyzed using an atomic absorption spectrophotometer with a graphite furnace (Spectra AA, Varian, Palo Alto, CA, USA) for Cd and Pb, and with a flame atomic spectrophotometer equipped with a deuterium lamp background correction (AAnalyst 100, PerkinElmer, Waltham, MA, USA) for Mg, Fe, Zn and Cu. Magnesium, Fe, Zn and Cu concentrations in the tissues were expressed in μg/g and Cd and Pb in ng/g on a wet weight (ww) basis. All the samples were run in batches, which included blanks; there was no evidence of any contamination in these blanks. The accuracy of the method was evaluated using the analysis of international standards (ERM ^®^—BB422 fish muscle for Mg, Fe, Zn, Cu and Cd and BB186 pig kidney for Pb). The concentrations found with the method used in this study fell into the certified uncertainty interval given by ERM, corresponding to a 95% confidence level.

The detection limits for flame atomic spectrophotometry were 0.04 μg/mL for Mg, 0.09 μg/mL for Fe, 0.04 μg/mL for Zn and 0.01 μg/mL for Cu. When the analyses were carried out using an atomic absorption spectrophotometer equipped with a graphite furnace, the detection limits were 0.6 ng/mL for Cd and 1.9 ng/mL for Pb.

### 4.3. Size Exclusion Chromatography

Six adult specimens of *V. crabro*, weighing 170–320 mg each, and two pools of three specimens of pupae or three of larvae, each pool weighing 800 mg, were homogenized in 5 volumes (*v/w*) of Tris-HCl 20 mM and 10 mM mercaptoethanol, pH 8.6, using an Ultraturrax (IKA, Staufen, Germany) homogenizer. The homogenate was centrifuged at 24,000 g for 30 min at 4 °C; the resulting supernatant was additionally centrifuged at the same speed (24,000 g) for 30 min at 4 °C. A volume of 1 mL cytosol was applied to a Sephadex G-75 chromatography column (0.9 × 90 cm) calibrated with rabbit Mt (Sigma) as a protein marker and eluted with the homogenizing buffer; the remaining part was stored at −80 °C for additional analysis. Fractions of 1.5 mL were collected and analyzed for Mg, Fe, Zn and Cu concentrations using the direct aspiration of the solution into the flame of atomic absorption spectrophotometer as described above. The pellets obtained from the centrifugation of the homogenate were digested in a microwave oven, as reported above, and Mg, Fe, Zn and Cu concentrations were determined using a flame atomic absorption spectrophotometer as reported in paragraph 4.2.

### 4.4. SDS–PAGE

In the supernatants and fractions obtained from size exclusion chromatography, the total proteins were determined by direct measurement of the absorbance at 280 nm (DeNovix DS-11 Series spectrophotometer, Wilmington, DE, USA). Fifteen µg of proteins were loaded onto 4–12% bis-Tris polyacrylamide gels (NuPAGE/Thermo Fisher Scientific, Waltham, MA, USA), and electrophoresis (PAGE) was carried out in an Xcell SureLock Mini-Cell with 2-(N-morpholino) ethanesulfonic acid buffer (MES; NuPAGE/Thermo Fisher Scientific, Waltham, Massachusetts, MA, USA), a running buffer at pH 7.3 containing sodium dodecyl sulfate (SDS). Each gel was also loaded with standard proteins of known molecular weight (SeeBlue™ Plus2 pre-stained protein standard, Thermo Fisher Scientific, Waltham, MA, USA). The electrophoresis system was connected to a power supply (Power Pack Basic—Bio-Rad, Hercules, CA, USA) with a constant voltage of 200 V for 40 min. The gels were stained with Coomassie G250 compatible with mass spectrometry analysis. After staining, each gel was digitalized by ChemiDocMP (Bio-Rad, Hercules, CA, USA), and pherograms were obtained using ImageLab 5.2.1 software (Bio-Rad, Hercules, California, CA, USA).

### 4.5. Protein Identification Using Mass Spectrometry

The most represented bands were manually cut and underwent “in-gel” tryptic digestion. Briefly, the bands were first destained with acetonitrile (ACN); the proteins were then reduced by 10 mM dithiothreitol (DTT) at 56 °C for 30 min and were subsequently alkylated using 55 mM iodoacetamide for 20 min in the dark. After drying, the proteins were digested with 12.5 ng/μL trypsin (Promega, Madison, USA) by incubation overnight at 37 °C. Subsequently, the peptides were extracted using a solution composed of 1% trifluoroacetic acid/50% ACN and were finally concentrated in a vacuum dryer (Eppendorf Concentrator Plus).

The dried digested samples were resuspended in 95% water/3% ACN/2% formic acid and analyzed using a UHPLC-MS QExactive™ system (Thermo Fisher Scientific, Reinach, Switzerland), composed of a UHPLC 3000 Ultimate System coupled to an ESI-QExactive™ Hybrid Quadrupole-Orbitrap™ mass spectrometer (LC-ESI-QO-MS/MS System). The separations were carried out on a ZORBAX RRHD Eclipse Plus C18 column (50 × 2.1 mm ID, 1.8 μm particle size, Agilent Technologies, Santa Clara, USA) with a mobile phase composed of 0.1% aqueous formic acid solution (A) and ACN (B), using the following gradient elution at a flow-rate of 0.3 mL/min: 0 to 3 min: isocratic at 2% (B); 3 to 21 min: linear gradient from 2 to 27% (B); 21 to 25 min: linear gradient from 27 to 90% (B); 25 to 28 min: isocratic at 90% (B); 28 to 28.1 min: linear gradient from 90 to 2% (B). An equilibration period of 6.9 min was interposed between each run. Nitrogen was used for spray stabilization, for collision-induced dissociation experiments in the higher energy collision dissociation (HCD) cell, and as damping gas in the C-trap. The ESI source operated in positive ionization mode, with a capillary voltage of 3.5 kV. The analyses were checked using Xcalibur™ software (version 29, build 2926).

**Figure 3:**
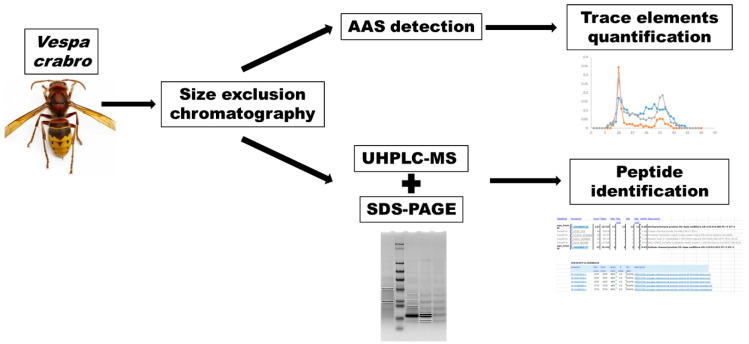
Schematic representation of the analyses performed on samples of *V. crabro*. AAS, atomic absorption spectrometry; SDS–PAGE, sodium dodecyl sulfate-polyacrylamide gel electrophoresis; UHPLC-MS, ultra high-performance liquid chromatography–mass spectrometry.

The raw mass data acquired, once converted into Mascot generic format using MsConvert (version 3.0.10730, ProteoWizard tools) were searched using the MASCOT search engine (version 2.7, http://mascot.cigs.unimo.it/mascot). Trembl and SwissProt, together with C-RAP protein databases, were selected for peptide sequence and contaminant searching, respectively, setting the following restriction parameters: trypsin as a proteolytic enzyme, max two missed cleavages; peptide mass tolerance ± 10 ppm (for precursor ions) and fragment mass tolerance ± 0.02 Da (for product ions); unrestricted protein mass; carbamidomethylation to cysteine residues as a fixed modification, and deamidated (NQ) and oxidated (M) methionine as variable modifications. Since the *V. crabro* protein database is not well annotated, a broader taxonomy, namely “Metazoa (Animals)”, was selected for identification to be based on sequence homology. Proteins with the highest score hits among MASCOT search results, identified with at least two or more significant peptides, were selected. Moreover, an automatic decoy database search was used to estimate the false discovery rate (FDR), setting the significant threshold to obtain an FDR < 1%. Figure 3 summarizes the analyses performed in *V. crabro*.

### 4.6. Statistical Analysis

The statistical analysis was carried out using statistical software (RStudio-1.2.1335 Statistical and R, R version 3.4.3). All data were evaluated using standard descriptive statistics and reported as mean ± standard deviation (SD). Normality was checked using the Shapiro–Wilk test. Variables (Fe, Mg, Cu, Zn, Cd, Pb) were compared among groups using nonparametric statistics. The Mann–Whitney U test was used to evaluate the differences between the species (*V. crabro* vs. *V. velutina*) and between pellets and cytosolic extracts. The Kruskal–Wallis rank-sum test and post hoc tests (Dunn test or Mann–Whitney U test) were used to evaluate the differences within each species among the body parts (head vs. thorax vs. abdomen) and among developmental stages in *V. crabro*. A *p*-value < 0.05 was considered significant.

## 5. Conclusions

In summary, the data in the present study provided a better understanding of hornet essential and non-essential element handling and a foundation for additional studies. In the authors’ opinion, hornets are an interesting animal model for discovering new information regarding the relationship between essential elements, in particular, Mg and Fe, and energy metabolism during flight. Proteome research has contributed to a new understanding regarding this topic, revealing the abundance of enzymes involved in carbohydrate catabolism in cytosolic extracts. This study also highlighted the changes in essential element speciation during metamorphosis. Finally, additional evidence was reported regarding the already accepted use of insects as bioindicators of environmental metal contamination.

The study of the metal metabolism in hornets and in other unconventional animals is a paradigm of how basic science, supported by molecular tools, can aid in shedding more light on common, but still not sufficiently explored, biochemical adaptations, and it should be encouraged in the future.

## Figures and Tables

**Table 1:** Element concentrations in the adult European hornet (*V. crabro*) and Asian yellow-legged hornet (*V. velutina*). Data are reported as mean ± standard deviation (SD), and are expressed in µg/g wet weight (ww) for Mg, Fe, Zn and Cu and in ng/g ww for Cd and Pb. For each metal, different capital letters indicate significant differences between species. For each metal, different lower cases indicate a significant difference among body parts within each species.

Species	Body Parts	Mg	Fe	Zn	Cu	Cd	Pb
*V. crabro*	whole body n = 60	378.16 ± 70.12 A	70.61 ± 18.02 A	35.62 ± 6.97 A	11.79 ± 2.10 A	44.92 ± 22.51 A	101.00 ± 94.67 A
*V. crabro*	head N = 10	365.40 ± 23.61 A a	23.78 ± 8.37 A a	28.82 ± 3.14 A a	6.68 ± 1.46 A a	39.63 ± 38.84 A ab	128.20 ± 2.54 A b
*V. crabro*	thorax N = 10	308.06 ± 28.95 A b	27.39 ± 11.82 A a	21.81 ± 2.18 A b	8.54 ± 1.40 A b	6.29 ± 0.40 A b	22.04 ± 2.89 A a
*V. crabro*	abdomen N = 10	394.38 ± 55.92 A a	71.60 ± 9.08 A b	46.93 ± 16.11 A c	11.96 ± 3.10 A c	65.54 ± 44.20 A a	80.56 ± 77.19 A ab
*V. velutina*	whole body n = 60	300.62 ± 58.69 B	46.66 ± 7.27 B	23.98 ± 3.94 B	12.67 ± 2.48 A	61.70 ± 25.27 A	30.91 ± 17.76 B
*V. velutina*	head N = 10	329.63 ± 6.25 B a	14.89 ± 0.61 B a	26.60 ± 1.47 A a	6.22 ± 0.67 A a	47.59 ± 27.53 A ab	37.71 ± 23.32 B a
*V. velutina*	thorax N = 10	324.71 ± 18.65 A a	28.31 ± 1.73 B b	20.02 ± 1.29 B b	9.54 ± 0.57 A b	11.91 ± 0.33 B a	11.78 ± 1.88 B a
*V. velutina*	abdomen N = 10	269.15 ± 32.27 B b	69.92 ± 8.90 A c	39.71 ± 8.63 A c	24.06 ± 2.63 B c	163.92 ± 33.98 A b	69.79 ± 22.60 A a

n = single specimen; N = number of pools of 3 specimens each.

**Table 2:** Element concentrations in the different developmental stages of the European hornet (*V. crabro*). Data are reported as mean ± standard deviation (SD), and are expressed in µg/g wet weight (ww) for Mg, Fe, Zn and Cu and in ng/g (ww) for Cd and Pb. For each metal, different lower cases indicate significant differences.

	Mg	Fe	Zn	Cu	Cd	Pb
Adult whole body n = 60	378.16 ± 70.12 a	70.61 ± 18.02 a	35.62 ± 6.97 a	11.79 ± 2.10 a	44.92 ± 22.51 a	101.00 ± 94.67 a
Pupa n = 30	264.94 ± 62.63 b	29.33 ± 7.17 b	13.56 ± 1.59 b	4.71 ± 0.67 b	7.56 ± 12.77 ab	15.55 ± 13.53 b
Larva n = 30	240.64 ± 67.61 b	38.01 ± 18.17 b	18.79 ± 8.44 b	5.85 ± 2.75 b	49.34 ± 44.78 b	10.78 ± 5.15 b
Queen n = 5	164.28 ± 26.12 c	41.21 ± 9.58 b	81.94 ± 60.74 a	12.57 ± 5.15 a	52.33 ± 11.68 ab	33.58 ± 33.96 a
Meconium n = 10	641.91 ± 89.85	251.26 ± 74.27	180.76 ± 111.79	53.56 ± 5.54	296.19 ± 46.28	141.14 ± 81.24

n = single specimen.

**Table 3:** Element distribution between pellets (P) and cytosolic extracts (CE) from the European hornet (*V. crabro*). Data are reported as mean ± standard deviation (n = 6 for adult specimens and n = 2 for larvae and pupae) and are expressed in percentages (%). For each metal, different capital letters indicate significant differences between pellets and cytosolic extracts.

	Mg		Fe		Zn		Cu	
	P	CE	P	CE	P	CE	P	CE
Larva	33 ± 2 A	67 ± 2 B	42 ± 8 A	58 ± 8 A	36 ± 1 A	64 ± 1 B	17 ± 5 A	83 ± 5 B
Pupa	41 ± 6 A	59 ± 6 B	84 ± 2 A	16 ± 2 B	53 ± 6 A	47 ± 6 A	39 ± 5 A	61 ± 5 B
Adult	87 ± 3 A	13 ± 3 B	72 ± 8 A	28 ± 8 B	47 ± 5 A	53 ± 5 A	56 ± 9 A	44 ± 9 A

**Table 4:** Protein identification in fractions from cytosolic extracts of larvae, pupae and adults of European hornet (*V. crabro*) using mass spectrometry.

Band ^1^	Entry Name ^2^	Protein Full Name	MW (Da) ^3^	Score ^4^	Sign. Pept.^5^	Sign Seq.^6^	Organism
1	A0A088ALS8	4-alpha-glucanotransferase	178,903	221	15	12	*Apis mellifera*
	A0A088A777	Sodium channel protein	292,184	17	2	2	*Apis mellifera*
2	A1IHL0	Alpha-glucosidase isozyme	66,623	52	2	1	*Apis cerana japonica*
	A0A088AUT8	Trehalase	77,540	37	2	2	*Apis mellifera*
	BIP_DROME	Endoplasmic reticulum chaperone BiP	72,330	59	3	3	*Drosophila melanogaster*
3	A0A088AHC8	Glyceraldehyde-3-phosphate dehydrogenase	36,146	192	13	8	*Apis mellifera*
	A0A088AUT2	Uncharacterized protein	1,067,315	68	7	7	*Apis mellifera*
	V9IJ78	Enolase	29,942	59	2	2	*Apis cerana*
	G6PI_DROME	Glucose-6-phosphate isomerase	62,585	52	3	3	*Drosophila melanogaster*
4	A0A088A4K0	Phosphoglycerate mutase 2	35,396	187	10	6	*Apis mellifera*
5	A0A088A933	Superoxide dismutase [Cu-Zn]	15,795	89	4	2	*Apis mellifera*
	A4ZXC4	Triosephosphate isomerase	26,939	30	2	2	*Apis mellifera*
6	A0A087ZYC6	Dynein light chain 2, cytoplasmic	10,465	57	3	3	*Apis mellifera*
7	A0A088AN20	Ubiquitin-like domain-containing protein	14,979	333	32	6	*Apis mellifera*

^1^ Number of the identified band as marked in Figure 2 ^2^ Protein entry name from the UniProt knowledge database. ^3^ Theoretical protein molecular mass. ^4^ The highest scores were obtained with the Mascot search engine. ^5^ Significant peptides: total number of significant peptides matching the identified proteins. ^6^ Significant sequences: total number of significant distinct sequences matching the identified proteins.

## Data Availability

The data presented in this study are available on request from the corresponding author.

## Supplementary Materials

Supplementary materials can be found at https://www.mdpi.com/1422-0067/22/1/228/s1. Table S1: The function and biological classification of the proteins identified in the chromatographic fractions obtained after gel filtration chromatography of cytosolic extracts of larvae, pupae and adults of European hornet using mass spectrometry. The biological processes and molecular functions according to the gene ontology (GO) and UniProt databases.

## Abbreviations

AAS	Atomic absorption spectrometry
ACN	Acetonitrile
ATP	Adenosine triphosphate
CE	Cytosolic extracts
DTT	Dithiothreitol
FDR	False discovery rate
GO	Gene ontology
GTP	Guanosine triphosphate
HCD	Higher energy collision dissociation
HMM	High molecular mass
LC-ESI-QO-MS	Liquid chromatography–electrospray ionization quadrupole orbitrap mass spectrometer
LMM	Low molecular mass
MES	2-(N-morpholino) ethanesulfonic acid buffer
Mts	Metallothioneins
SD	Standard deviation
SOD	Superoxide dismutase
SDS–PAGE	Sodium dodecyl sulfate-polyacrylamide gel electrophoresis
UHPLC-MS	Ultra high-performance liquid chromatography mass spectrometer
ww	Wet weight
