# Supplementary material for: Essential (Mg, Fe, Zn and Cu) and Non-Essential (Cd and Pb) Elements in Predatory Insects (Vespa crabro and Vespa velutina): A Molecular Perspective
Source: Int J Mol Sci. 2020 Dec 28;22(1):228. doi: 10.3390/ijms22010228 (PMC7797950; doi:10.3390/ijms22010228)
Supplement: Supplementary file 1 [file ijms-22-00228-s001.pdf]

Supplementary Materials

Entry Name [a]	Protein Full Name	Organism	Biological Process	Molecular Function	Cellular component
A1IHL0	Alpha-glucosidase isozyme	Apis cerana japonica	NA	hydrolase activity; maltose alpha-glucosidase activity	membrane; integral component of membrane
A0A087ZYC6	dynein light chain 2	Apis mellifera	microtubule-based process	motor activity	cytoskeleton; cytoplasm
BIP_DROME	Endoplasmic reticulum chaperone BiP	Drosophila melanogaster	cellular response to unfolded protein; ubiquitin-dependent ERAD pathway; sleep; RNA interference; response to unfolded protein; response to endoplasmic reticulum stress; protein refolding; intestinal stem cell homeostasis; endoplasmic reticulum unfolded protein response; chaperone cofactor-dependent protein refolding	ATPase activity; ATP binding; heat shock protein binding; unfolded protein binding; protein folding chaperone; misfolded protein binding	Endoplasmic reticulum
V9IJ78	Enolase	Apis cerana	glycolytic process	magnesium ion binding; phosphopyruvate hydratase activity	cytosol; phosphopyruvate hydratase complex
A0A088A7S9	Gal_mutarotas_2 domain-containing protein	Apis mellifera	carbohydrate metabolic process	hydrolase activity, carbohydrate binding; hydrolyzing O-glycosyl compounds	NA
G6PI_DROME	Glucose-6-phosphate isomerase	Drosophila melanogaster	gluconeogenesis; glucose 6-phosphate metabolic process; glucose homeostasis; glycolytic process	glucose-6-phosphate isomerase activity; monosaccharide binding	cytosol
A0A088AHC8	Glyceraldehyde-3-phosphate dehydrogenase	Apis mellifera	glucose metabolic process; glycolytic process	glyceraldehyde-3-phosphate dehydrogenase (NAD+) (phosphorylating) activity; NADP binding; NAD binding	NA
A0A088A9P3	Importin-4-like isoform X1	Apis mellifera	protein import to nucleus	Ran GTPase binding	cytoplasm
A0A088A4K0	phosphoglycerate mutase 2	Apis mellifera	glycolytic process	biphosphoglycerato mutase acitvity; phosphoglycerato mutase acitvity;	NA
A0A087ZNT2	Profilin	Apis mellifera	NA	actin binding	cytoskeleton; cytoplasm
A0A088A777	Sodium channel protein	Apis mellifera	regulation of ion transmembrane transport	voltage-gated ion channel activity; voltage-gated sodium channel activity	cell membrane; multi-pass membrane protein
A0A088A933	Superoxide dismutase [Cu-Zn]	Apis mellifera	NA	metal ion binding; superoxide dismutase activity	NA
A0A088AUT8	Trehalase	Apis mellifera	trehalose metabolic process	alpha,alpha trehalase activity	membrane; integral component of membrane
